# Vital Signs: Communication Between Health Professionals and Their Patients About Alcohol Use — 44 States and the District of Columbia, 2011

**Published:** 2014-01-10

**Authors:** Lela R. McKnight-Eily, Yong Liu, Robert D. Brewer, Dafna Kanny, Hua Lu, Clark H. Denny, Lina Balluz, Janet Collins

**Affiliations:** 1 Div of Population Health, National Center for Chronic Disease Prevention and Health Promotion, CDC; 2 Div of Birth Defects and Developmental Disabilities, National Center for Birth Defects and Developmental Disabilities, CDC; 3 Div Of Environmental Hazards & Health Effects, National Center for Environmental Health, CDC; 4 Div of Nutrition, Physical Activity, and Obesity, National Center for Chronic Disease Prevention and Health Promotion, CDC

## Abstract

**Introduction:**

Excessive alcohol use accounted for an estimated 88,000 deaths in the United States each year during 2006–2010, and $224 billion in economic costs in 2006. Since 2004, the U.S. Preventive Services Task Force (USPSTF) has recommended alcohol misuse screening and behavioral counseling (also known as alcohol screening and brief intervention [ASBI]) for adults to address excessive alcohol use; however, little is known about the prevalence of its implementation. ASBI will also be covered by many health insurance plans because of the Affordable Care Act.

**Methods:**

CDC analyzed Behavioral Risk Factor Surveillance System (BRFSS) data from a question added to surveys in 44 states and the District of Columbia (DC) from August 1 to December 31, 2011, about patient-reported communication with a health professional about alcohol. Elements of ASBI are traditionally delivered via conversation. Weighted state-level prevalence estimates of this communication were generated for 166,753 U.S. adults aged ≥18 years by selected demographic characteristics and drinking behaviors.

**Results:**

The prevalence of ever discussing alcohol use with a health professional was 15.7% among U.S. adults overall, 17.4% among current drinkers, and 25.4% among binge drinkers. It was most prevalent among those aged 18–24 years (27.9%). However, only 13.4% of binge drinkers reported discussing alcohol use with a health professional in the past year, and only 34.9% of those who reported binge drinking ≥10 times in the past month had ever discussed alcohol with a health professional. State-level estimates of communication about alcohol ranged from 8.7% in Kansas to 25.5% in DC.

**Conclusions:**

Only one of six U.S. adults, including binge drinkers, reported ever discussing alcohol consumption with a health professional, despite public health efforts to increase ASBI implementation.

**Implications for Public Health Practice:**

Increased implementation of ASBI, including systems-level changes such as integration into electronic health records processes, might reduce excessive alcohol consumption and the harms related to it. Routine surveillance of ASBI by states and communities might support monitoring and increasing its implementation.

## Introduction

Excessive alcohol use accounted for an estimated 88,000 deaths and 2.5 million years of potential life lost* in the United States each year during 2006–2010 (1), and an estimated $224 billion in economic costs in 2006 (2). Excessive alcohol use is associated with increases in the chances of heart disease, breast cancer, sexually transmitted diseases, unintended pregnancy, fetal alcohol spectrum disorders, sudden infant death syndrome, motor-vehicle crashes, violence, suicide, and many other health problems (3). It includes binge drinking, exceeding weekly limits (for men, 15 or more on average in a week; for women, eight or more on average per week); and any use by pregnant women or persons aged <21 years.† A standard drink is considered 12 ounces of 5% beer, 5 ounces of 12% wine, or 1.5 ounces (a shot) of 80-proof distilled spirits or liquor (e.g., gin, rum, vodka, or whiskey). In 2011, binge drinking was reported by 18.3% of U.S. adults (38 million persons) surveyed through the Behavioral Risk Factor Surveillance System (BRFSS),§ who reported doing so an average of approximately four times a month and consuming approximately eight drinks per occasion on average (4).

In 2005, the National Institute on Alcohol Abuse and Alcoholism (NIAAA) published updated ASBI clinical guidelines (5) to include screening for the number of days of binge-level alcohol consumption in the past year among adults.¶ ASBI traditionally involves a conversation between a health professional and patient to screen using a standardized set of questions (can be by form) and/or discuss the results of screening for excessive alcohol use. For those who screen positive, the brief counseling intervention involves a dialogue about motivations and steps to reduce drinking because of health dangers, based on consumption guidelines and the patient’s medical status. The small number of patients who are alcoholics or have a severe alcohol use disorder should be referred for specialized treatment. Since 2004, the U.S. Preventive Services Task Force (USPSTF) has recommended alcohol misuse screening and behavioral counseling (also known as alcohol screening and brief intervention or ASBI) for all adults in primary care, including pregnant women, to address excessive alcohol use (6).

This review of evidence indicated that brief (6–15 minutes) intervention sessions were effective in significantly reducing weekly alcohol consumption (by 3.6 fewer drinks/week for adults) and binge level episodes (reported by 12% fewer participants), and increasing adherence to recommended drinking limits (achieved by 11% more participants). Further, effects can last for years and show improvement in health-care utilization outcomes including fewer hospital days and lower costs. However, despite evidence of effectiveness and longstanding recommendations for ASBI implementation, limited information is available to assess aspects such as communication between a health professional and patient. This analysis is based on data from the responses of U.S. adults to a single question about their dialogue with a health professional about alcohol use. This question was initially added to the BRFSS as a part of a clinical preventive services optional module included on some state surveys during 1996–1999.

## Methods

BRFSS is an annual, state-based, random-digit–dialed telephone survey of noninstitutionalized U.S. adults aged ≥18 years that collects information on health conditions and risk behaviors, including alcohol use (7). From August 1 through December 31, 2011, all BRFSS respondents in 44 states and DC were read the following lead-in statement: “The next question is about counseling services related to prevention that you might have received from a doctor, nurse, or other health professional.” Respondents were then asked: “Has a doctor or other health professional ever talked with you about alcohol use?” as an emerging core question. Respondents who answered affirmatively were asked when the talk occurred (e.g., within the past year). Responses were stratified by selected sociodemographic variables and drinking behavior (current drinking, binge drinking, and frequency of binge drinking).** In 2011, the overall median survey response rate†† was 49.7% (range: 33.8%–64.1%); for states included in this report, the range was 33.8%–61.4%). A total of 166,753 respondents (including 20,711 cellular telephone respondents) were included in the analysis. Weighted prevalence estimates were derived using statistical software. Two-tailed t-tests were used to assess statistical significance (p<0.05). Comparisons are statistically significant unless otherwise noted.

## Results

The overall weighted prevalence of ever having dialogue with a health professional about alcohol use was 15.7% (Table 1), and past year prevalence was 7.6%. Ever discussing alcohol use was significantly higher for men (19.0%) than women (12.5%) and similar among pregnant (17.3%) and nonpregnant (16.9%) women aged 18–44 years. It was more common among those aged 18–24 (27.9%) and declined significantly with increasing age. The prevalence of ever having dialogue about alcohol use with a health professional was significantly higher for Hispanics (22.5%) and non-Hispanic blacks (19.4%) than for non-Hispanic whites (13.7%) and other non-Hispanics (15.8%). Respondents without a high school diploma (19.9%) and those with an annual household income of <$25,000 (20.2%) had a significantly higher prevalence than those with higher education and income levels. Prevalence was also significantly higher among those unable to work (29.2%) than among the employed (14.6%) or retired (10.2%) and was higher among persons without health insurance coverage (20.0%) than those with health insurance (14.8%). Finally, this dialogue was significantly more common among never-married respondents (23.6%) and members of an unmarried couple (19.9%) than among married respondents (12.6%), and divorced, widowed, or separated persons (15.0%).

**FIGURE 1:**
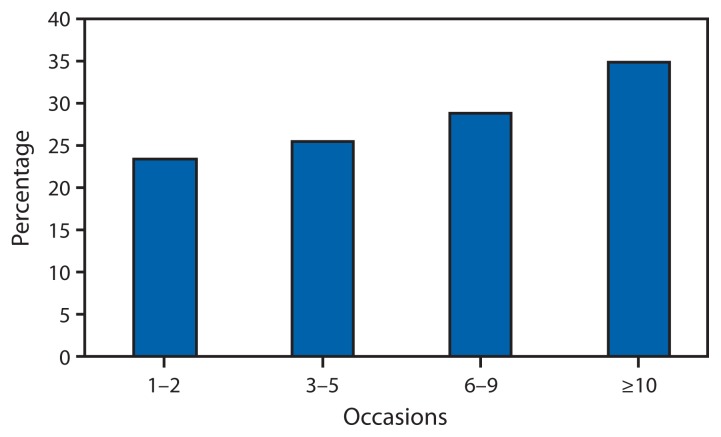
Weighted prevalence of ever discussing alcohol use with a doctor or other health professional among U.S. adult binge drinkers, by binge drinking frequency in the past month — Behavioral Risk Factor Surveillance System, 44 states and the District of Columbia, 2011

The prevalence of ever having been spoken with about alcohol by a health professional was 17.4% among current drinkers and 13.5% among nondrinkers (Table 1). Prevalence among binge drinkers (25.4%) was approximately twice that of non-binge drinkers (13.5%), and increased significantly with the number of binge drinking episodes, ranging from 23.6% (95% confidence interval [CI]: 19.4–28.4) among those reporting one to two episodes to 34.9% (95% CI: 29.7–40.4) among those reporting ≥10 episodes during the past 30 days (Figure 1).

**FIGURE 2:**
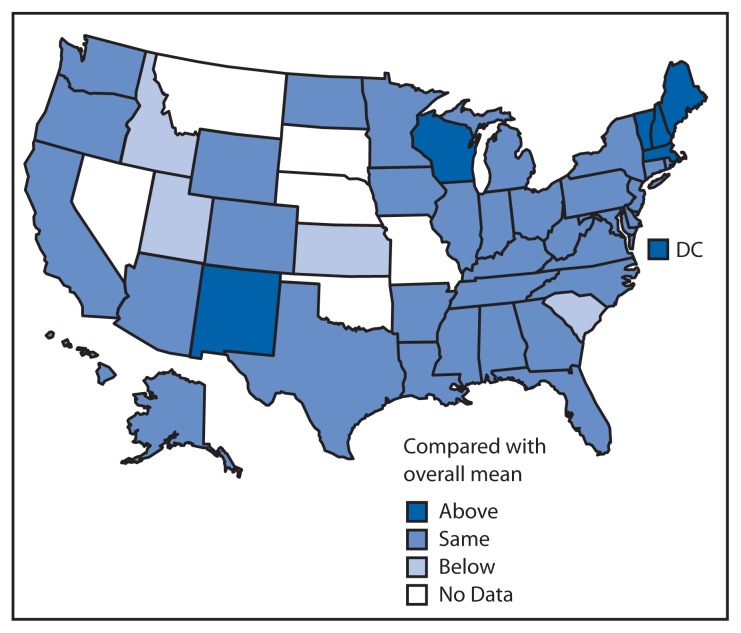
Age-adjusted prevalence of ever discussing alcohol use with a doctor or other health professional among U.S. adults, in comparison with overall mean estimate — Behavioral Risk Factor Surveillance System, 44 states and the District of Columbia, August 1–December 31, 2011

Overall, state-based estimates of ever having communication with a health professional about alcohol ranged from 8.7% in Kansas to 25.5% in DC, with the highest concentration in the northeastern states and lowest in the middle southern states (Table 2). However, most state prevalence estimates were not significantly different from the overall mean prevalence for all participating states (Figure 2).

## Conclusions and Comment

The results of this analysis indicate that in 2011, only one in six U.S. adults overall, one in five current drinkers, and one in four binge drinkers in 44 states and DC reported ever discussing alcohol use with a doctor or other health professional. Further, 65.1% of those who reported binge drinking ≥10 times in the past month had never had this dialogue. These findings are consistent with previous reports: in 1997, only 23% of U.S. adult binge drinkers in 10 states reported being spoken with about alcohol use on the BRFSS, and in a 2011 study, only 14% of young adults who reported exceeding alcohol consumption guidelines and visiting a doctor were asked about their alcohol use (8,9).

Variations in participant recall of their interactions with their health professionals or differences in the offering of certain clinical preventive services in primary care environments might have affected these communications. Nonetheless, the overall prevalence of health professionals talking with patients regarding alcohol use is still very low, based on findings from this and similar reports, despite USPSTF recommendations for all adults in primary care to be screened and receive brief counseling, if warranted. A survey of U.S. adults in 12 metropolitan areas found that preventive care interventions, including screening for problem drinking, were underused. Only 54.9% of the recommended percentage of preventive care, 18.3% of recommended counseling or education, and 10.5% of recommended care was received for alcohol dependence (10). Even among trauma and hepatitis patients, documented screening for problem drinking during hospitalization was low (11).

The findings in this report are subject to at least five limitations. First, BRFSS data are based on self-report and dependent on respondent recall of dialogue with a health professional, which can vary based on the time since the patient’s last visit or other factors that could have affected patient recall, thus resulting in underreporting. Second, respondents were asked to report only whether they “talked with” a health professional about their alcohol consumption, not whether they reported their alcohol consumption in some other manner (e.g., on a patient history form) or if they were actually screened or received an intervention. However, NIAAA recommends that regardless of the screening method used, health professionals should discuss alcohol use with all patients. For patients who drink, but not excessively, the discussion (or a patient brochure) should focus on maximum drinking limits and situations when less drinking, or no drinking (as for pregnant women, persons aged <21 years, and those with health conditions or taking medications that interact negatively with alcohol) is advisable. The Dietary Guidelines for Americans also recommend that adults who drink only do so in moderation, defined as up to one drink a day for women and two for men, and not starting to drink more for possible health benefits (12). NIAAA provides guidelines for discussions for persons who screen positive for excessive drinking (which includes binge drinking) in its Clinicians’ Guide (5). The data also did not include information on the extent of the alcohol intervention and changes in drinking behavior that might result. Third, the data used in this analysis were only collected in 44 states and DC and for a portion of the year (i.e., August 1–December 31, 2011); therefore, prevalence estimates might not be representative of the entire United States. Fourth, BRFSS does not collect information by landline from persons living in institutional settings (e.g., on military bases), and the prevalence of talking with a health professional about alcohol consumption might be different in these groups. Finally, the survey median response rate was 49.7%, raising the possibility of response bias.

ASBI was ranked by the National Commission on Prevention Priorities as one of the five most effective clinical preventive services (along with blood pressure control, low cholesterol, breast cancer screening, and annual influenza vaccination), based on the clinically preventable burden of disease and intervention cost effectiveness (13). The Affordable Care Act of 2010 allows for health insurance coverage for ASBI,§§ billing codes are available for ASBI services,¶¶ and model benefit plan language for ASBI has been developed for use in public and private health insurance plans.*** ASBI has also been endorsed by national health organizations, and implementation guidelines have been published by NIAAA, the World Health Organization, and CDC (5,14,15). Further, the Substance Abuse and Mental Health Services Administration has funded grantees and is collaborating with the Centers for Medicare and Medicaid Services to educate health-care providers about Medicare billing and insurance coverage for alcohol SBI services.††† Additional federal efforts include requiring states with expanded Medicaid to cover a set of preventive services, including alcohol screening and counseling through the Affordable Care Act (16), and studying the best means for implementing alcohol screening and counseling at federally qualified health centers (17).

Barriers to screening and counseling identified by health-care providers include lack of time, training, and self-efficacy; discomfort discussing the topic; perceived difficulty working with substance use patients; skepticism of treatment effectiveness; patient resistance; and lack of insurance coverage (18). These and other implementation barriers might be addressed through health professional organizations working to increase training and education for health providers and working with employers to understand the benefits of including ASBI as a part of their health plans. Systems-level changes by health plans and insurers, such as adopting recommended guidelines, including ASBI as a part of standard service that all patients receive, providing insurance coverage, and incentives for the delivery of ASBI, also might address barriers and improve implementation (18,19). A key aspect of routinizing alcohol screening and counseling as standard practice in medical practice includes ensuring that staff comprehend that most patients who drink too much will only require brief counseling, not specialized treatment. Support from key staff members and stakeholders, including the development and testing of an implementation plan, and training on the use of guidelines, is also needed (20). Finally, the use of a variety of health professionals (e.g., doctors, nurses, clinical social workers) to screen all patients, including women who are or could be pregnant (should be advised not to drink at all), and intervene with those who screen positive for drinking too much through the use of approved guidelines (5,6,18), can also address provider concerns, particularly about time and efficacy. Screening and counseling can also occur in several settings, including emergency departments, trauma centers, and OB/GYN practices (20).

**Key Points**

- In 2011, only about one in six U.S. adults and one in four binge drinkers in 44 states and the District of Columbia (DC) reported that a health professional had ever discussed alcohol use with them. This has changed very little in the past 15 years.
- Excessive alcohol use, including binge drinking, is responsible for approximately 88,000 deaths in the United States each year, and cost the nation an estimated $224 billion in 2006.
- Alcohol screening and brief intervention (ASBI) or counseling is an effective strategy that health professionals can use to help their adult patients, including pregnant women, reduce excessive alcohol use.
- ASBI traditionally involves a conversation between a health-care provider and patient to screen or interpret the results of screening for excessive alcohol use. For those who screen positive, the intervention involves a dialogue about motivations and steps to reduce drinking, based on consumption guidelines and the patient’s medical status.
- Discussing alcohol consumption was most prevalent among persons aged 18–24 years (27.9%) and those who reported binge drinking ≥10 times in the past month (34.9%).
- The prevalence of health-care professional communication about alcohol ranged from 8.7% in Kansas to 25.5% in DC.
- Increased implementation of ASBI-related services could help reduce excessive alcohol consumption and the harms related to it.
- Routine surveillance of ASBI-related services could support its implementation and monitoring of progress.
- Additional information is available at http://www.cdc.gov/vitalsigns.

The Community Preventive Services Task Force has recommended several community level interventions to reduce excessive alcohol use,§§§ including electronic screening and brief intervention (e.g., use of computers, telephones, or mobile devices to deliver components of ASBI), which have reduced peak consumption by 25% among binge drinkers in reviewed studies and might help to reduce implementation barriers in clinical settings. Providing physicians and other health professionals with prompts and feedback regarding ASBI might also be an effective strategy. For example, ASBI is being considered for inclusion as a meaningful use measure¶¶¶ in the electronic health records process which, if included and then implemented, might increase its use.

## Figures and Tables

**TABLE 1:** Weighted prevalence of discussing alcohol use with a doctor or other health professional among U.S. adults, by sociodemographic characteristics — Behavioral Risk Factor Surveillance System, 44 states and the District of Columbia, August 1–December 31, 2011

Characteristic	Unweighted No.	Talked with about alcohol use			

		Ever		During past year	
	
		%	(95% CI)	%	(95% CI)
**Total**	**166,753**	**15.7**	**(15.0–16.4)**	**7.6**	**(6.9–8.2)**
**Sex**					
Men	64,836	19.0	(17.9–20.3)	9.2	(8.0–10.5)
Women	101,917	12.5	(12.0–13.1)	6.0	(5.7–6.4)
**Pregnancy status (females aged 18–44 yrs only)**					
Yes	998	17.3	(13.4–21.6)	11.9	(9.0–15.6)
No	23,996	16.9	(15.9–17.9)	8.2	(7.6–8.9)
**Age (yrs)**					
18–24	6,529	27.9	(24.2–32.1)	15.9	(12.0–20.6)
25–34	15,411	17.1	(16.0–18.1)	7.8	(7.1–8.6)
35–44	21,333	14.6	(13.7–15.5)	6.5	(6.0–7.2)
45–64	68,414	14.6	(13.9–15.2)	6.7	(6.3–7.1)
≥65	53,525	9.3	(8.8–9.8)	4.2	(3.9–4.6)
**Race/Ethnicity**					
White, non-Hispanic	130,722	13.7	(13.3–14.1)	6.2	(6.0–6.5)
Black, non-Hispanic	14,844	19.4	(17.9–21.0)	10.6	(9.4–11.9)
Hispanic	10,379	22.5	(19.1–26.3)	11.9	(8.6–16.4)
Other, non-Hispanic*	8,916	15.8	(14.2–17.5)	6.9	(5.9–8.0)
**Education**					
Less than high school diploma	14,326	19.9	(18.4–21.5)	9.6	(8.4–10.9)
High school diploma or equivalent	47,456	16.7	(15.7–17.6)	7.8	(7.3–8.5)
Some college	44,601	15.4	(14.6–16.2)	6.7	(6.2–7.3)
College graduate	60,017	13.4	(12.8–13.9)	6.6	(6.2–7.0)
**Employment status**					
Employed	81,353	14.6	(14.1–15.1)	6.8	(6.5–7.2)
Unemployed	10,042	19.7	(18.0–21.5)	9.3	(8.3–10.4)
Retired	48,177	10.2	(9.6–10.8)	4.6	(4.3–5.0)
Unable to work	12,224	29.2	(22.5–36.8)	15.1	(8.5–25.4)
Homemaker or student	14,418	18.6	(17.1–20.1)	9.7	(8.6–10.9)
**Marital status**					
Married	88,982	12.6	(12.2–13.1)	6.1	(5.8–6.4)
Divorced, widowed, separated	50,181	15.0	(14.0–16.0)	6.1	(5.7–6.6)
Never married	22,756	23.6	(21.2–26.1)	12.4	(10.0–15.3)
Member of unmarried couple	4,121	19.9	(17.3–22.8)	8.7	(7.2–10.5)
**Annual household income**					
<$25,000	42,675	20.2	(18.2–22.4)	9.8	(7.8–12.2)
$25,000 to <$50,000	37,920	14.4	(13.6–15.3)	6.3	(5.7–6.9)
$50,000 to <$75,000	22,854	13.7	(12.8–14.7)	5.9	(5.4–6.6)
≥$75,000	40,466	13.6	(12.9–14.3)	7.3	(6.7–7.8)
**Health insurance coverage**					
Yes	148,057	14.8	(14.4–15.3)	7.3	7.3(7.0–7.6)
No	18,200	20.0	(17.0–23.3)	9.0	(6.1–13.0)
**Current alcohol consumption**					
Yes	85,870	17.4	(16.4–18.4)	9.0	(8.0–10.0)
No	79,762	13.5	(12.8–14.2)	5.7	(5.3–6.2)
**Binge drinking** †					
Yes	20,993	25.4	(22.8–28.3)	13.4	(10.6–16.7)
No	143,788	13.5	(13.0–13.9)	6.2	(5.9–6.5)

**Abbreviation:** CI = confidence interval.

* Includes Asian, Native Hawaiian or other Pacific Islander, American Indian or Alaskan Native, other race, and multiracial.

† Binge drinking is defined as four or more drinks on at least one occasion during the past 30 days for women or five or more drinks on at least one occasion during the past 30 days for men.

**TABLE 2:** Age-adjusted prevalence of ever discussing alcohol use with a doctor or other health professional among U.S. adults, in comparison with overall mean estimate — Behavioral Risk Factor Surveillance System, 44 states and the District of Columbia, August 1–December 31, 2011

State	%	(95% CI)
State average	15.8	(15.2–16.5)
**Above state average** *		
District of Columbia	25.5	(22.5–28.7)
Maine	19.6	(17.9–21.4)
Massachusetts	21.2	(19.7–22.8)
New Hampshire	21.1	(18.9–23.5)
New Mexico	18.7	(16.8–20.7)
Rhode Island	19.1	(16.9–21.7)
Vermont	20.0	(17.9–22.3)
Wisconsin	21.6	(18.3–25.2)
**On state average** †		
Alabama	15.5	(13.6–17.7)
Alaska	16.6	(14.0–19.6)
Arizona	15.4	(12.9–18.4)
Arkansas	12.7	(10.2–15.7)
California	15.4	(14.3–16.6)
Colorado	15.3	(13.8–17.0)
Connecticut	17.6	(15.4–20.1)
Delaware	16.7	(14.5–19.3)
Florida	18.7	(12.9–26.5)
Georgia	14.9	(13.4–16.7)
Hawaii	17.8	(15.8–20.1)
Illinois	14.4	(12.4–16.5)
Indiana	16.5	(14.6–18.6)
Iowa	15.3	(13.6–17.3)
Kentucky	14.3	(12.4–16.3)
Louisiana	14.4	(12.8–16.2)
Maryland	16.8	(15.0–18.9)
Michigan	14.9	(13.2–16.9)
Minnesota	14.6	(13.3–16.0)
Mississippi	13.9	(12.3–15.8)
New Jersey	14.4	(12.9–15.9)
New York	18.2	(15.9–20.8)
North Carolina	14.6	(12.9–16.4)
North Dakota	16.6	(14.2–19.3)
Ohio	16.5	(14.6–18.5)
Oregon	14.7	(12.5–17.3)
Pennsylvania	15.2	(13.6–16.9)
Tennessee	13.9	(11.0–17.4)
Texas	15.2	(13.5–17.0)
Virginia	14.5	(12.3–17.1)
Washington	16.0	(14.1–18.0)
West Virginia	14.1	(12.3–16.2)
Wyoming	14.9	(13.1–16.9)
**Below state average** §		
Idaho	11.9	(10.1–14.0)
Kansas	8.7	(7.8–9.8)
South Carolina	13.6	(12.2–15.2)
Utah	11.8	(10.6–13.1)

**Abbreviation:** CI = confidence interval.

* Statistically above the stage average at p<0.05, based on two-tailed t-tests.

† No statistical difference from the state average at p<0.05, based on two-tailed t-tests.

§ Statistically below the state average at p<0.05, based on two-tailed t-tests.

## Footnotes

* YPLL for 2006–2010 were estimated using the Alcohol-Related Disease Impact (ARDI) application using death and life expectancy data from the National Vital Statistics System. Additional information is available at http://apps.nccd.cdc.gov/dach_ardi/default/default.aspx.

† Additional information available at http://www.cdc.gov/alcohol/faqs.htm#excessivealcohol.

§ Additional information available at http://apps.nccd.cdc.gov/brfss.

¶ The NIAAA-recommended screening question for heavy drinking days is as follows: “How many times in the past year have you had five or more drinks in a day (for men) or four or more drinks in a day (for women)?”

** Binge drinkers were defined as respondents who consumed four or more drinks per occasion during the preceding 30 days for women and five or more drinks for men. Frequency of binge drinking was calculated based on the total number of binge drinking episodes during the past 30 days. An occasion is generally defined as 2–3 hours.

†† Response rates for BRFSS are calculated using standards set by the American Association of Public Opinion Research response rate formula no. 4, available at http://www.aapor.org/standard_definitions2.htm. The response rate is the number of respondents who completed the survey as a proportion of all eligible and likely eligible persons. The cooperation rate median and range was 54.6% to 89.0% (median: 77.0%). The cooperation rate is the percentage of persons who completed interviews among all eligible persons who were contacted. Additional information is available at http://www.cdc.gov/brfss/pdf/2011_summary_data_quality_report.pdf.

§§ The Patient Protection and Affordable Care Act of 2010 requires that nongrandfathered private health plans provide coverage without cost-sharing for services that have in effect an “A” or “B” recommendation from the USPSTF. Because the USPSTF issued a “B” recommendation for ASBI in adults aged ≥18 years, this must be covered by such plans, Section 1001 of the Patient Protection and Affordable Care Act, Public Law 111-148, 2010. Available at http://www.gpo.gov/fdsys/pkg/PLAW-111publ148/html/PLAW-111publ148.htm.

¶¶ Dedicated Healthcare Common Procedure Coding System (HCPCS) codes H0049 and H0050, which health-care providers can use to bill Medicare and Medicaid for ASBI services, and Current Procedural Terminology (CPT) codes 99408 and 99409.

*** Additional information available at https://www.healthcare.gov/what-are-my-preventive-care-benefits/#. http://www.businessgrouphealth.org/pub/f2f59214-2354-d714-5198-3a8968092869.

††† Additional information available at http://www.samhsa.gov/prevention/sbirt.

§§§ The Community Preventive Services Task Force has recommended several community-based strategies for preventing excessive alcohol consumption (e.g., increasing alcohol taxes, regulating alcohol outlet density, and holding alcohol retailers liable for harms related to the sale of alcoholic beverages to minors and intoxicated patrons (dram shop liability). Additional information available at http://www.thecommunityguide.org/alcohol/index.html.

¶¶¶ Additional information available at http://www.healthit.gov/policy-researchers-implementers/meaningful-use.
